# Overexpression of GPX3, a potential biomarker for diagnosis and prognosis of breast cancer, inhibits progression of breast cancer cells in vitro

**DOI:** 10.1186/s12935-020-01466-7

**Published:** 2020-08-06

**Authors:** Weiyang Lou, Bisha Ding, Shuqian Wang, Peifen Fu

**Affiliations:** Department of Breast Surgery, First Affiliated Hospital, College of Medicine, Zhejiang University, 79 QingChun Road, Hangzhou, 310003 Zhejiang China

**Keywords:** Glutathione peroxidase 3 (GPX3), Breast cancer, Diagnosis, Prognosis, Biomarker

## Abstract

**Background:**

Growing evidence has demonstrated that glutathione peroxidases (GPXs) family genes play critical roles in onset and progression of human cancer. However, a systematic study regarding expression, diagnostic and prognostic values, and function of GPXs family genes in breast cancer remains absent.

**Materials and methods:**

Several databases were employed to perform in silico analyses for GPXs family genes. qRT-PCR, western blot and immunohistochemistry staining were introduced to validate GPX3 expression in breast cancer. The functions of GPX3 in breast cancer cells were successively determined.

**Results:**

By combination of receiver operating characteristic (ROC) curve analysis, survival analysis and expression analysis, GPX3 was considered as a potential tumor suppressor and a promising diagnostic/prognostic biomarker in breast cancer. Next, low expression of GPX3 was confirmed in breast cancer cells and tissues when compared with corresponding normal controls. Overexpression of GPX3 markedly suppressed proliferation, colony formation, migration and invasion of breast cancer in vitro. Moreover, two potential mechanisms responsible for GPX3 downregulation in breast cancer, including hypermethylation of GPX3 promoter and release of hsa-miR-324-5p inhibition.

**Conclusions:**

Collectively, we demonstrate that GPX3 is markedly downregulated in breast cancer, possesses significant diagnostic and prognostic values and attenuated in vitro growth and metastasis of breast cancer.

## Background

Breast cancer is the most common diagnosed women’s malignant tumor and also the second leading cause of cancer-related deaths in women worldwide [1, 2]. Despite a variety of advancements have been achieved in diagnosis and therapy, the total outcome of patients with breast cancer remains unsatisfactory. Thus, developing effective therapeutic targets and promising biomarkers for diagnosis and prognosis prediction is very meaningful to improve prognosis of breast cancer.

Glutathione peroxidases (GPXs), consisting of eight members (GPX1-8), are ubiquitously expressed proteins that catalyze the reduction of hydrogen peroxides and organic hydroperoxides by glutathione [3]. GPX family members have been well demonstrated to be frequently aberrantly expressed and are also closely linked to progression of diverse types of human cancer, including kidney cancer [4], pancreatic cancer [5], hepatocellular carcinoma [6], cervical cancer [7] and gastric cancer [8]. However, a comprehensive study about expression, function, diagnostic and prognostic values of GPXs family in breast cancer remain absent.

In this study, we first assessed the roles of GPXs family genes in predicting diagnosis and prognosis of breast cancer and then determined the mRNA and protein expression of GPXs family genes in breast cancer using bioinformatic analysis. Next, the low expression of GPX3 was detected in breast cancer cells and tissues. Subsequently, the function of GPX3 in breast cancer cell growth and metastasis was also investigated. Finally, we explored the potential detailed mechanisms responsible for GPX3 downregulation in breast cancer.

## Materials and methods

### ROC curve analysis

Using TCGA breast cancer and normal breast expression data, the diagnostic values of GPXs family genes were evaluated by ROC curve as we previously described [9]. P-value < 0.05 was considered as statistically significant.

### Kaplan–Meier-plotter database analysis

Kaplan–Meier-plotter database (http://kmplot.com/analysis/), which is capable to access the effect of 54,000 genes on survival in 21 cancer types, including breast cancer, was employed to perform survival analysis for GPXs family genes and miRNAs in breast cancer [10]. Logrank P-value < 0.05 was considered as significant.

### GEPIA database analysis

GEPIA database (http://gepia.cancer-pku.cn/index.html), a newly developed interactive web server for analyzing the RNA sequencing expression data of 9736 tumors and 8587 normal samples from the TCGA and GTEx projects, was used to determine mRNA expression profile of GPXs family genes in breast cancer [11]. P-value < 0.05 was considered as statistical significance.

### Oncomine database analysis

Oncomine database (https://www.oncomine.org/), which is a cancer microarray database and integrated data-mining platform, was also utilized to analyze mRNA expression of GPXs family genes in breast cancer [12, 13]. Fold change (FC) > 1.5, P-value < 0.05 and a gene rank in top 10% were set as the thresholds for selecting the included datasets.

### UALCAN database analysis

The protein expression levels of GPXs family genes in breast cancer were assessed using UALCAN database (http://ualcan.path.uab.edu/index.html), which is a comprehensive, user-friendly and interactive web resource for analyzing cancer OMICS data [14]. UALCAN database was also introduced to determine the promoter methylation level of GPX3 in breast cancer. P-value < 0.05 of statistical analysis was considered to have significant differences.

### starBase database analysis

starBase database (http://starbase.sysu.edu.cn/index.php), an open-source platform for investigating miRNA-associated studies, was used to predict the upstream binding miRNAs of GPX3 [15, 16]. The correlation of GPX3 with miRNA in breast cancer and miRNA expression level in breast cancer were also assessed by starBase database. P-value < 0.05 was considered as statistical significance.

### Cell lines and clinical tissues

The human breast cancer cell lines MCF-7 and MDA-MB-231 and normal breast cell line MCF-10A were purchased from Shanghai Institute of Biological Science, Chinese Academy of Sciences (Shanghai, China). 59 breast cancer tissues and 59 matched normal tissues were obtained from 59 patients with breast cancer, who received surgical resection in the First Affiliated Hospital of Zhejiang University, College of Medicine (Hangzhou, China). This study was approved by the ethics committee of the First Affiliated Hospital of Zhejiang University, College of Medicine.

### RNA isolation and qRT-PCR

Total RNA was isolated from breast cancer cells and tissues by Trizol reagent (Invitrogen, USA). qRT-PCR was employed to detect GPX3 mRNA expression in breast cancer as we previously described [17]. GPX3 expression was normalized to GAPDH by the method of 2^−ddCt^. The sequences of primers used in this study: GPX3 forward primer: 5′-GAGCTTGCACCATTCGGTCT-3′; GPX3 reverse primer: 5′-GGGTAGGAAGGATCTCTGAGTTC-3′; GAPDH forward primer: 5′-AATGGACAACTGGTCGTGGAC-3′; GAPDH reverse primer: 5′-CCCTCCAGGGGATCTGTTTG-3′.

### Protein extraction and western blot

Protein of breast cancer cells was extracted using RIPA buffer (Beyotime, China) supplemented with protease and phosphatase inhibitors (Thermo Scientific, USA). Western blot was performed as previously described [18]. The primary antibodies of GPX3 (1:1000) and GAPDH (1:1000) were purchased from Abcam, and anti-rabbit peroxidase conjugated secondary antibody was purchased from Sigma (1:5000). GPX3 band density was normalized to GAPDH and quantified by ImageJ software.

### Immunohistochemistry (IHC) analysis

IHC was utilized to analyze the protein expression of GPX3 in breast cancer tissues and matched normal breast tissues as we previously reported [19].

### Establishment of stably-overexpressed cell

Full length of GPX3 was first amplified, after which the PCR product was cloned into pcDNA3.1-PURO vector digested with *BamH1* and *XhoI*. GPX3-overexpressed plasmid was transfected into breast cancer cells using Lipofectamine™ 3000 (Invitrogen, USA) according to the manufactures’ instruction. Then, stably-overexpressed cell was screened using puromycin (2 μg/mL).

### CCK-8 assay

2500 stably-overexpressed cells were seeded into 96-well plates, and cultured for varied period (24, 48, 72 and 96 h). At the culture end of each time point, 20 μl CCK-8 solution was added into each well and incubated for another 4 h at 37 °C. Finally, the optical density (OD) value at 450 nm of each well was determined by a microplate reader.

### Colony formation assay

1000 stably-overexpressed cells were seeded into six-well plates, and cultured for 2 weeks. At the end of culture, the plates were washed using phosphate buffered saline (PBS) for two times. Next, the plates were fixed in methanol for 15 min and stained with 0.1% crystal violet solution for another 10 min. Finally, the visible colonies of each well were counted.

### Wound healing assay

Wound healing assay was introduced to detect the migrated ability of breast cancer cells. 40 × 10^4^ stably-overexpressed cells were seeded into six-well plates. When the cells were grown to 100% confluence, a wound cross was made using a micropipette tip. Photographs were then taken through a microscopy immediately or 24 h after wounding.

### Transwell invasion assay

Cell invasion was determined by Transwell invasion assay. Briefly, transwell inserts were firstly coated with Matrigel (BD, USA). Then, 10 × 10^4^ stably-overexpressed cells suspended in 0.2 mL serum-free medium were added into inserts. And 0.6 mL medium containing 20% FBS was added to the lower compartment as a chemoattractant. After culturing for 48 h, the cells on the upper membrane were carefully removed using a cotton bud and cells on the lower surface were fixed with methanol for 15 min and successively stained with 0.1% crystal violet solution for 10 min. Photographs were then taken through a microscopy.

### Statistical analysis

Statistical analysis of bioinformatic analysis was performed by online databases as mentioned above. The results of experimental data were shown as mean ± SD. Student’s *t*-test was used to assess differences between two groups. The diagnostic value was determined by ROC curve analysis. A two-tailed value of P < 0.05 was considered as statistically significant.

## Results

### The diagnostic and prognostic values of GPXs family genes in breast cancer

**Fig. 1:**
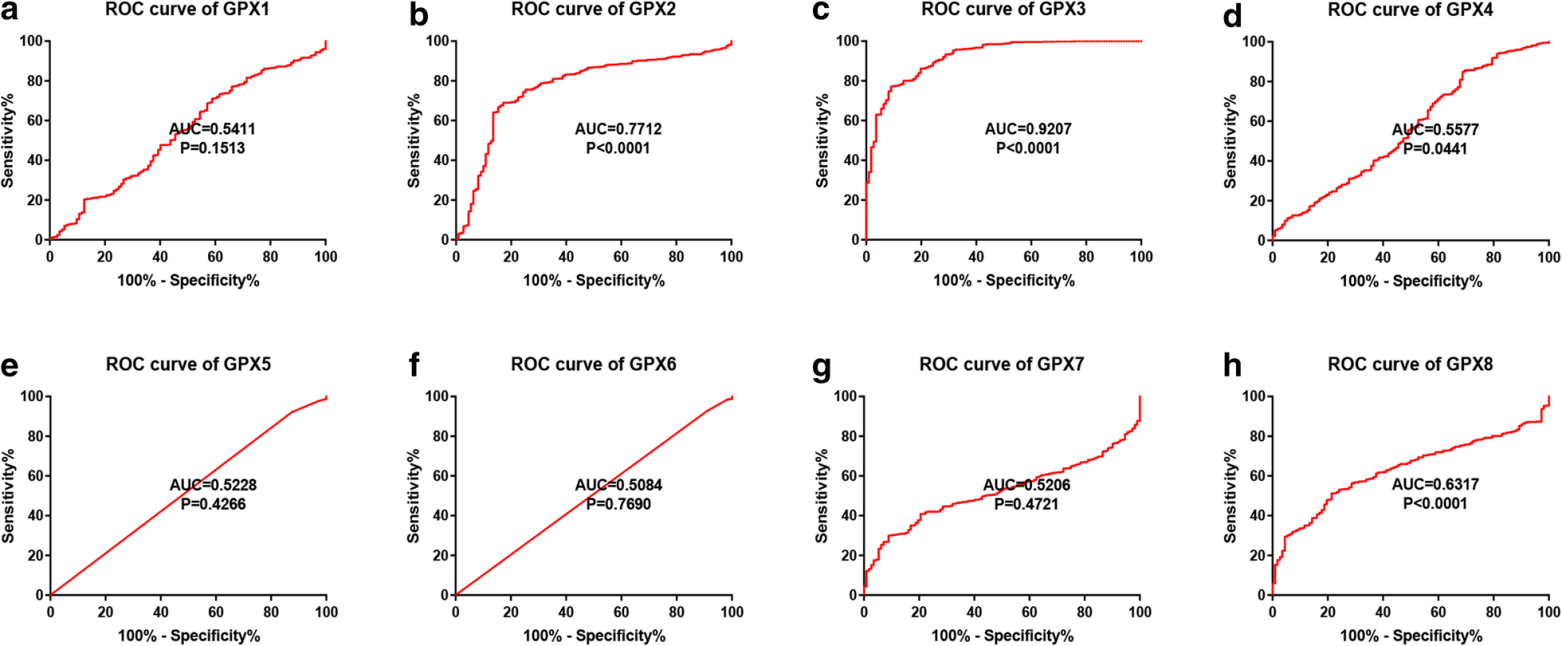
The diagnostic values of GPXs family genes in breast cancer using ROC curve analysis. **a** GPX1. **b** GPX2. **c** GPX3. **d** GPX4. **e** GPX5. **f** GPX6. **g** GPX7. **h** GPX8

**Fig. 2:**
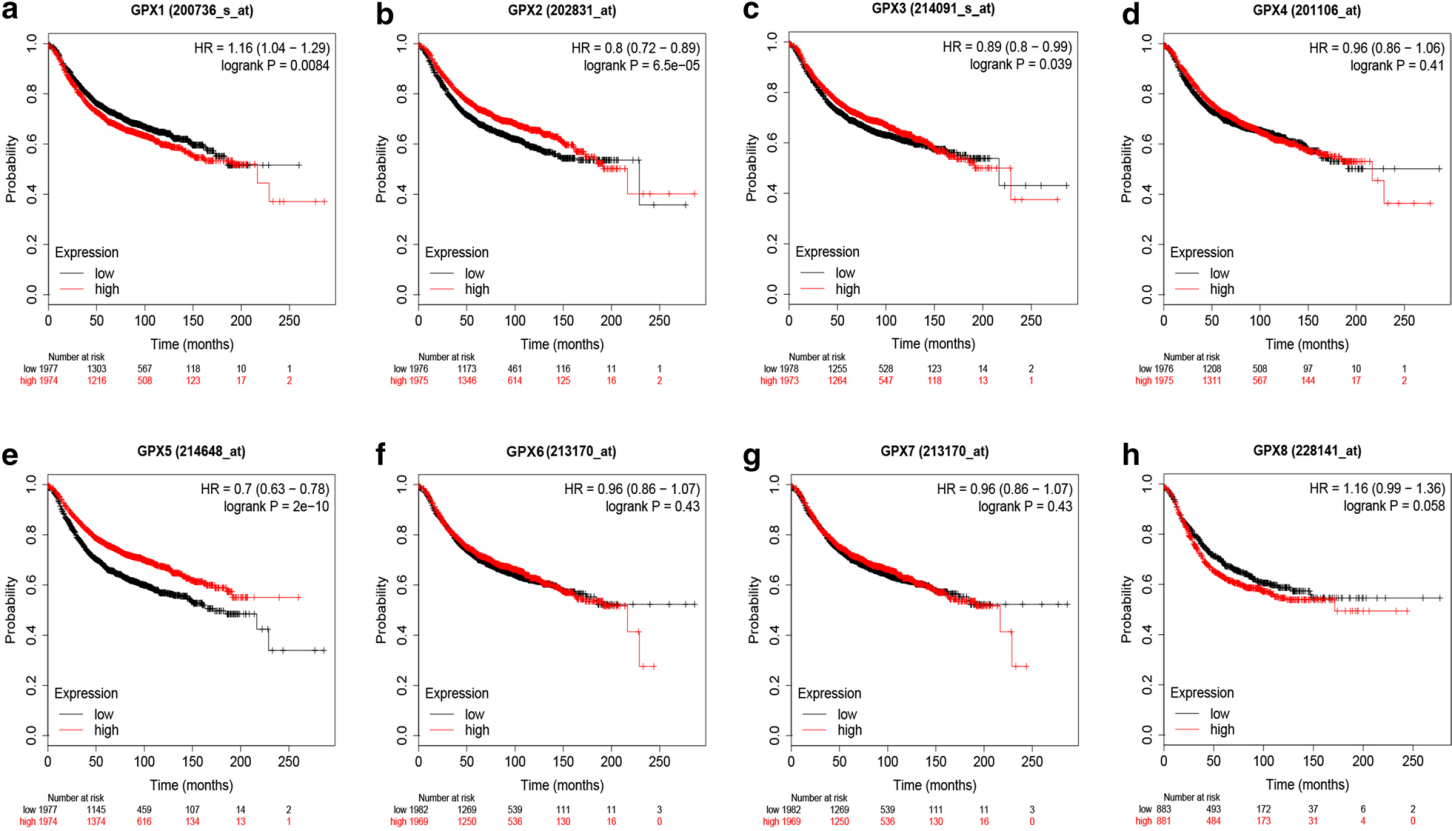
The prognostic values of GPXs family genes in breast cancer determined by Kaplan–Meier plotter database. **a** GPX1. **b** GPX2. **c** GPX3. **d** GPX4. **e** GPX5. **f** GPX6. **g** GPX7. **h** GPX8

To explore if the expression of GPXs family genes possesses significant diagnostic values in patients with breast cancer, receiver operating characteristic (ROC) curve analysis was employed based on breast cancer data from TCGA database (Fig. 1). As shown in Fig. 1, four GPXs family genes had the significant ability to distinguish breast cancer tissues from normal breast tissues, including GPX2, GPX3, GPX4 and GPX8. However, the other four GPXs family genes (GPX1, GPX5, GPX6 and GPX7) showed no statistical diagnostic values in breast cancer. Notably, these findings suggested that GPX3 was the most potential diagnostic biomarker for patients with breast cancer, with the Area Under Curve (AUC) value being equal to 0.9207. Next, we investigated the prognostic values of GPXs family genes in breast cancer using Kaplan–Meier-plotter database (Fig. 2). Increased expression of GPX1 (Fig. 2a) indicated poor prognosis of breast cancer. Breast cancer patients with higher expression of GPX2 (Fig. 2b), GPX3 (Fig. 2c) or GPX5 (Fig. 2e) had better prognosis. GPX4, GPX6 and GPX7 had no significant predictive values for prognosis of breast cancer. All these findings together indicated that only GPX2 and GPX3 possessed significant diagnostic and prognostic values for breast cancer.

### The expression levels of GPXs family genes in breast cancer

**Fig. 3:**
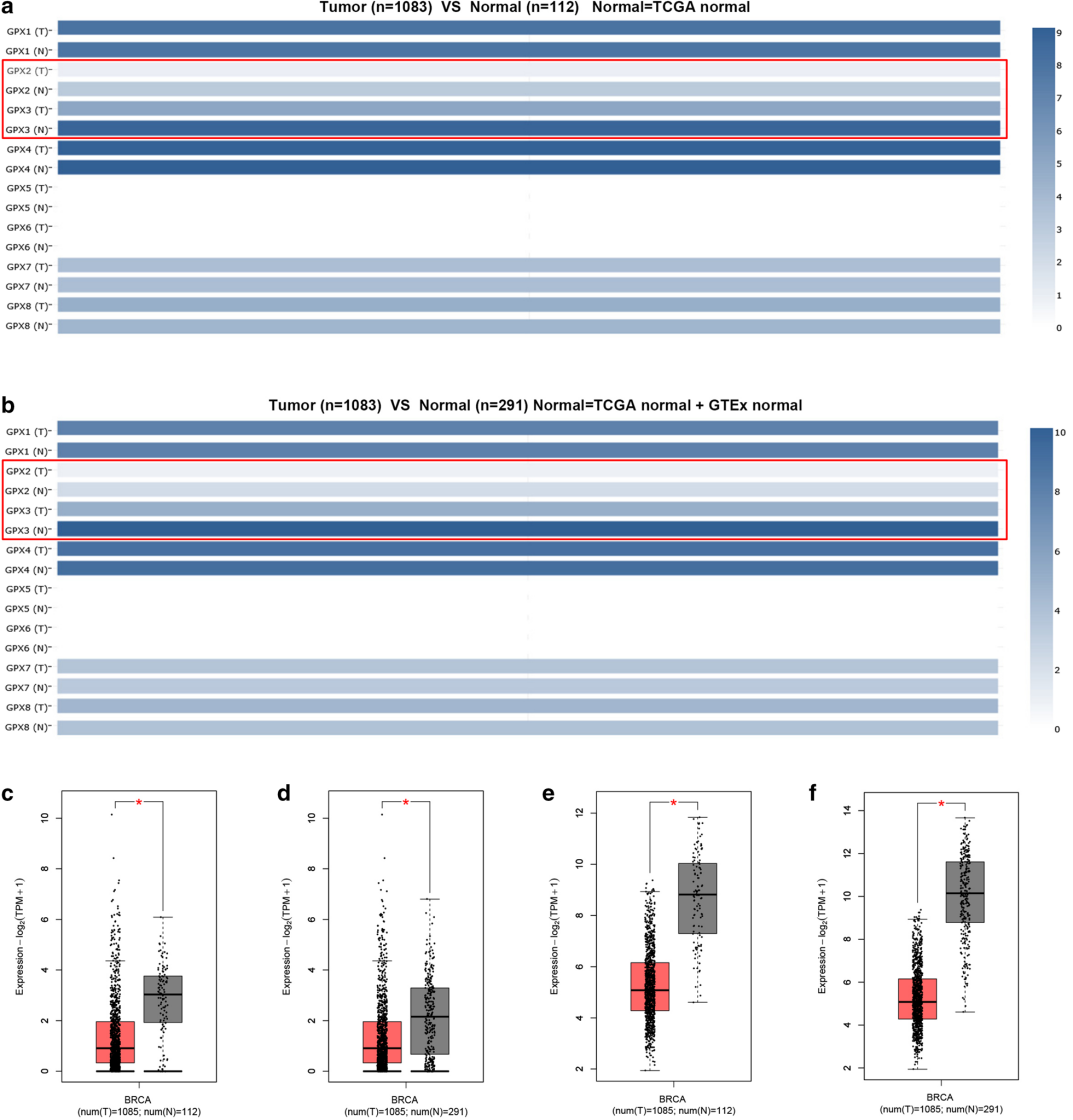
The mRNA expression of GPXs family genes in breast cancer determined by GEPIA database. **a** The mRNA expression profile of GPXs family genes in breast cancer tissues compared with TCGA normal breast tissues. **b** The mRNA expression profile of GPXs family genes in breast cancer tissues compared with TCGA and GTEx normal breast tissues. **c**, **d** GPX2 was significantly downregulated in breast cancer. **e**, **f** GPX3 was significantly downregulated in breast cancer. *P < 0.05

**Fig. 4:**
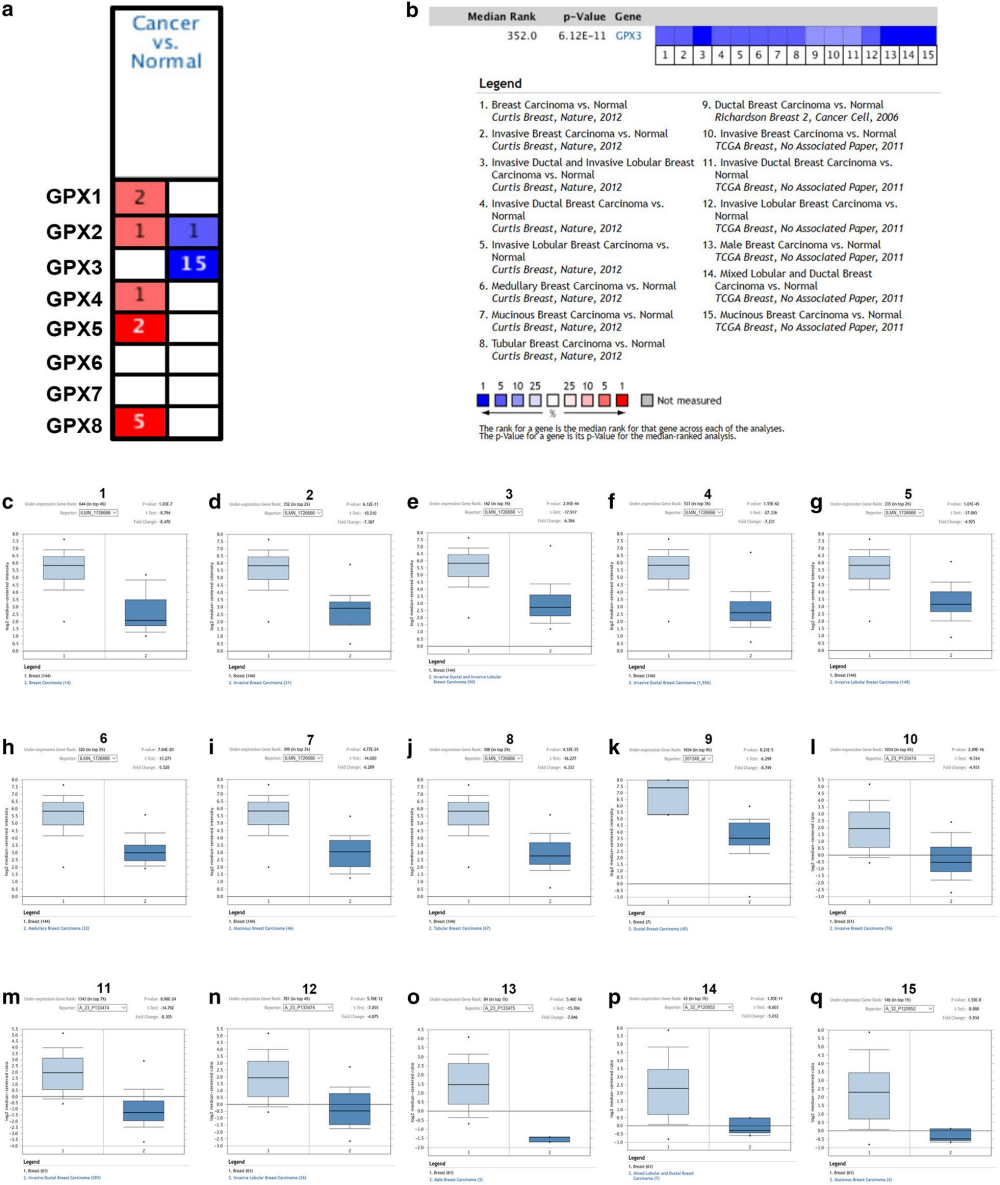
The mRNA expression of GPXs family genes in breast cancer determined by Oncomine database. **a** The mRNA expression of GPXs family genes in breast cancer. **b** Meta-analysis for the 15 included GPX3-associated datasets in breast cancer. **c**–**q** The mRNA expression of GPX3 was markedly downregulated in breast cancer in 15 included GPX3-assocaited datasets

**Fig. 5:**
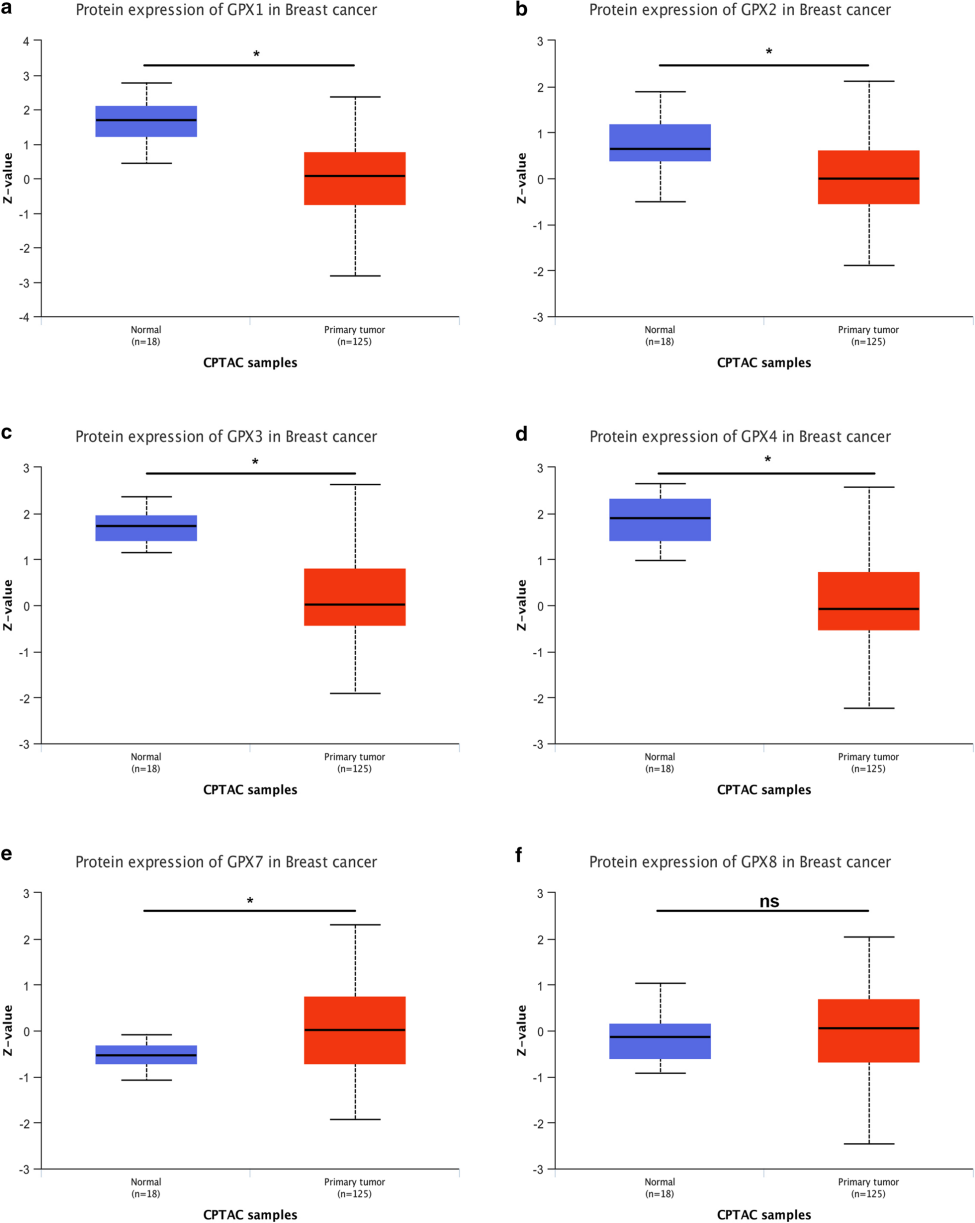
The protein expression of GPXs family genes in breast cancer detected by UALCAN database. **a** GPX1. **b** GPX2. **c** GPX3. **d** GPX4. **e** GPX7. **f** GPX8. *P < 0.05

**Fig. 6:**
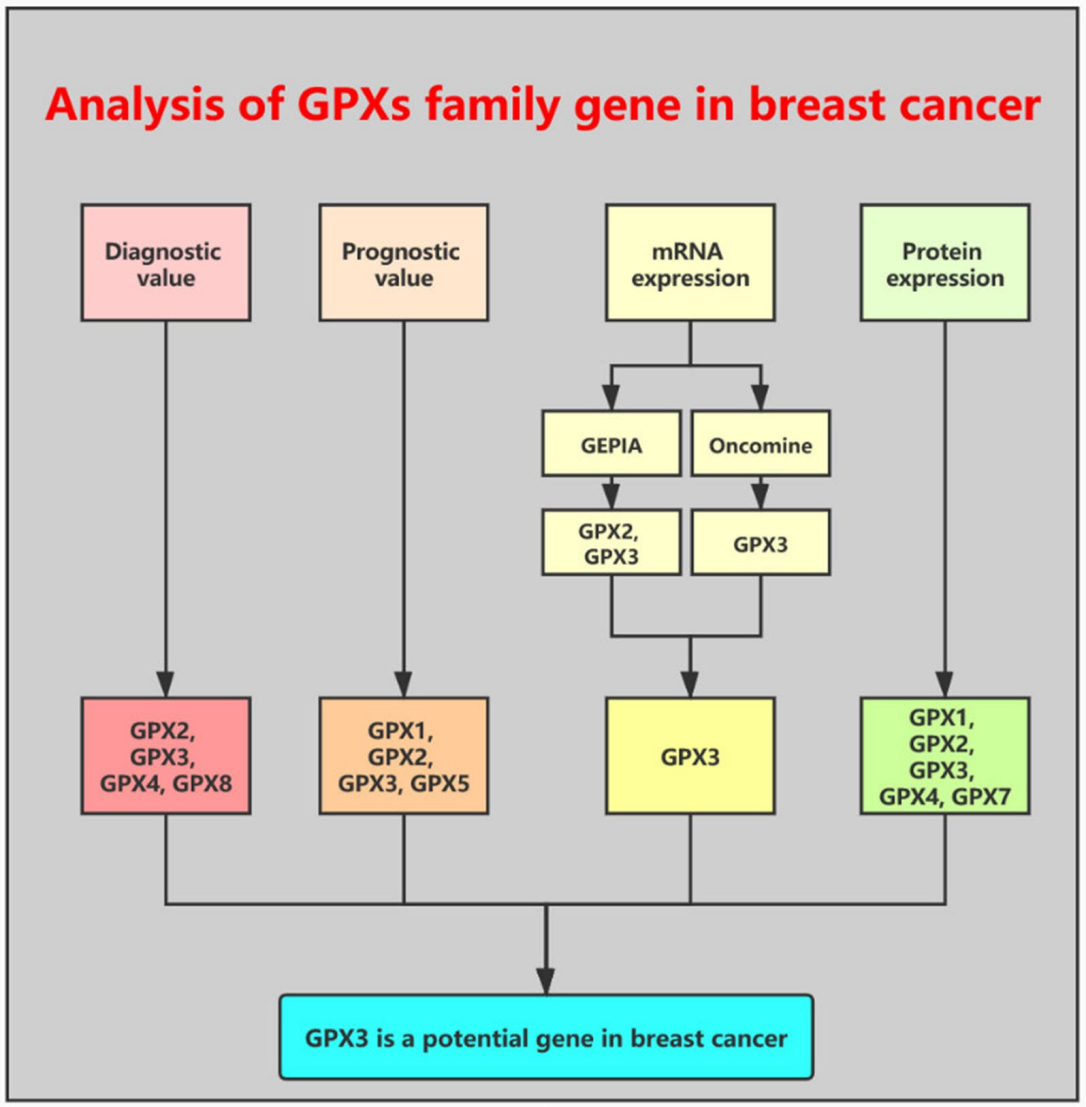
The visual flow-process diagram of this study

Next, we further studied the expression levels of GPXs family genes in breast cancer. First of all, TCGA and GTEx databases were introduced to mine the mRNA expression of 8 GPXs family genes in breast cancer. The mRNA expression profile of GPXs family was shown in Fig. 3a (TCGA tumor tissues compared with TCGA normal tissues) and Fig. 3b (TCGA tumor tissues compared with TCGA normal tissues and GTEx normal tissues). We found that GPX2 and GPX3 were significantly downregulated in breast cancer (Fig. 3c–f). Next, Oncomine database was used to further analyze mRNA expression of GPXs family genes in breast cancer (Fig. 4a). We performed meta-analysis for 15 included studies about GPX3, and found that GPX3 mRNA expression was markedly decreased in breast cancer (Fig. 4b). The downregulation of GPX3 mRNA expression in breast cancer of the 15 GPX3-associated studies was presented in Fig. 4c–q. However, we found that GPX2 was not significantly downregulated in breast cancer. Subsequently, CPTAC database was utilized to assess the protein expression of GPXs family genes in breast cancer (Fig. 5). The results revealed that GPX1, GPX2, GPX3 and GPX4 protein levels were markedly decreased in breast cancer when compared with normal controls. GPX7 protein expression in breast cancer was significantly increased. GPX8 showed no statistical difference between breast cancer tissues and normal tissues. And GPX5 and GPX6 were not found in CPTAC. Taken together, GPX3 was the most potential one among all GPXs family genes in breast cancer and was selected for following research (Fig. 6).

### The expression level of GPX3 was confirmed in breast cancer and negatively correlated with tumor progression

**Fig. 7:**
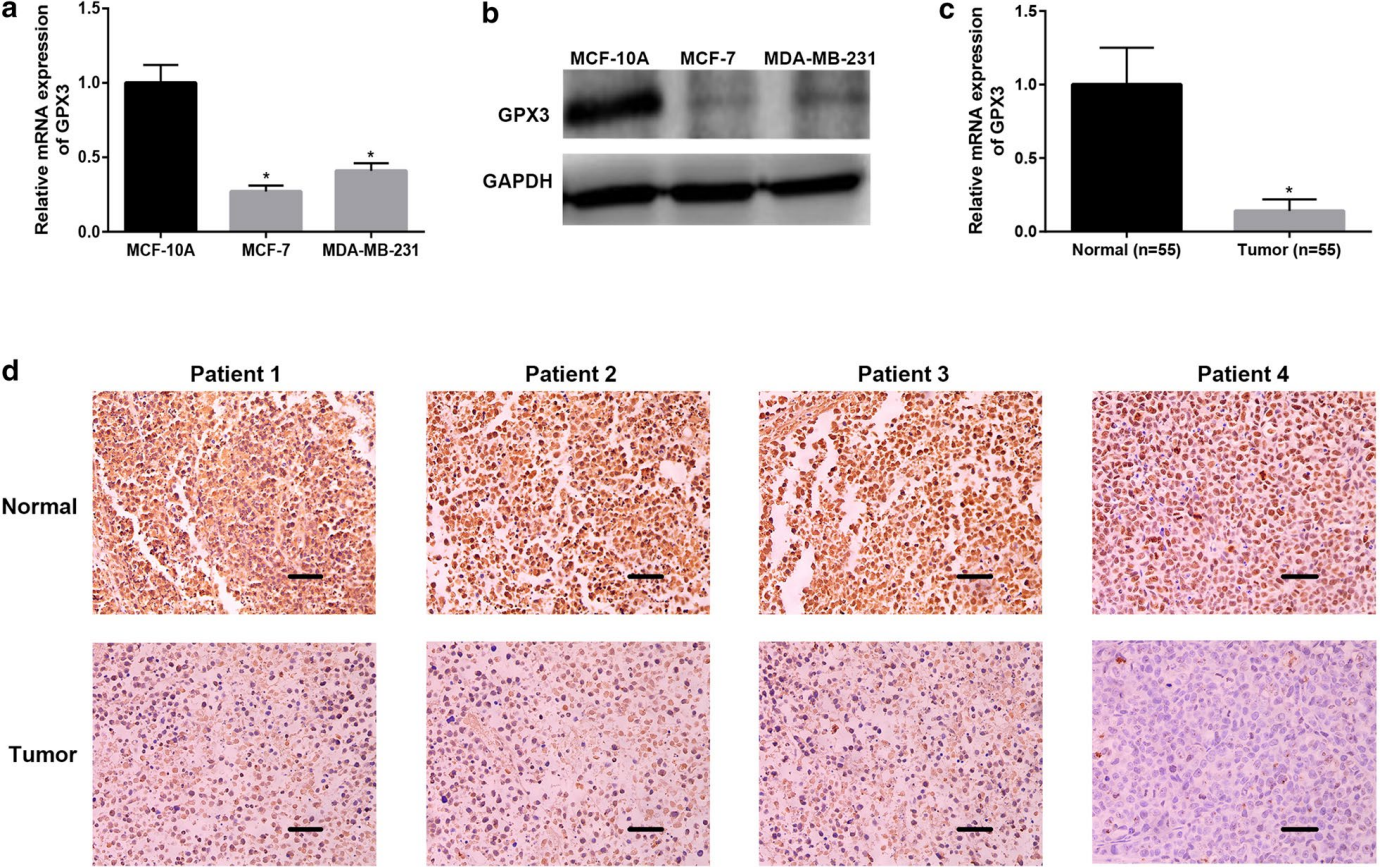
The expression levels of GPX3 in breast cancer cells and tissues. The mRNA (**a**) and protein (**b**) expression of GPX3 in breast cancer cells was significantly lower than that in normal breast cell. **c** The mRNA expression of GPX3 was markedly decreased in breast cancer tissues compared with matched normal breast tissues. **d** IHC analysis of GPX3 expression levels in normal breast tissues and breast cancer tissues. Bar scale: 150 um; *P < 0.05

To further validate the results from in silico analysis, we detected the mRNA and protein expression levels of GPX3 in breast cancer cells and tissues. As presented in Fig. 7a, b, GPX3 mRNA and protein were significantly downregulated in two breast cancer cells, MCF-7 and MDA-MB-231, when compared with normal cell, MCF-10A. We also found that GPX3 mRNA expression in breast cancer tissues was much lower than that in adjacent matched normal tissues (Fig. 7c). The protein expression of GPX3 was also detected using immunohistochemistry (IHC) analysis. The results showed that GPX3 protein expression was significantly decreased in breast cancer tissues (Fig. 7d). Collectively, GPX3 mRNA and protein expression levels were significantly downregulated in breast cancer, which was identical with the bioinformatic analytic results. Furthermore, Chi square test revealed that low expression of GPX3 was significantly negatively correlated with ER/PR expression and positively linked to tumor size, histopathological grade and lymph node metastasis (Table 1). All these findings showed that GPX3 was negatively correlated with progression of breast cancer and might function as a tumor suppressor in breast cancer.

**Table 1:** Correlation of GPX3 expression with various clinicopathological features in breast cancer

Features	Cases	Breast cancer		
		Low expression	High expression	P-value
Age				
≤ 50	22	7	15	0.7284
> 50	33	12	21	
Tumor size				
≤ 5	31	9	22	0.0289
> 5	24	14	10	
Lymph node metastasis				
Present	20	17	3	< 0.0001
Absent	35	5	30	
Histopathological grade				
I–II	41	12	29	0.0137
III	14	10	4	
ER status				
Positive	31	8	23	0.0012
Negative	24	17	7	
PR status				
Positive	36	10	26	< 0.0001
Negative	19	17	2	
HER2 status				
Positive	15	13	2	0.0629
Negative	40	22	18	

### GPX3 overexpression suppressed proliferation and colony formation of breast cancer cells

**Fig. 8:**
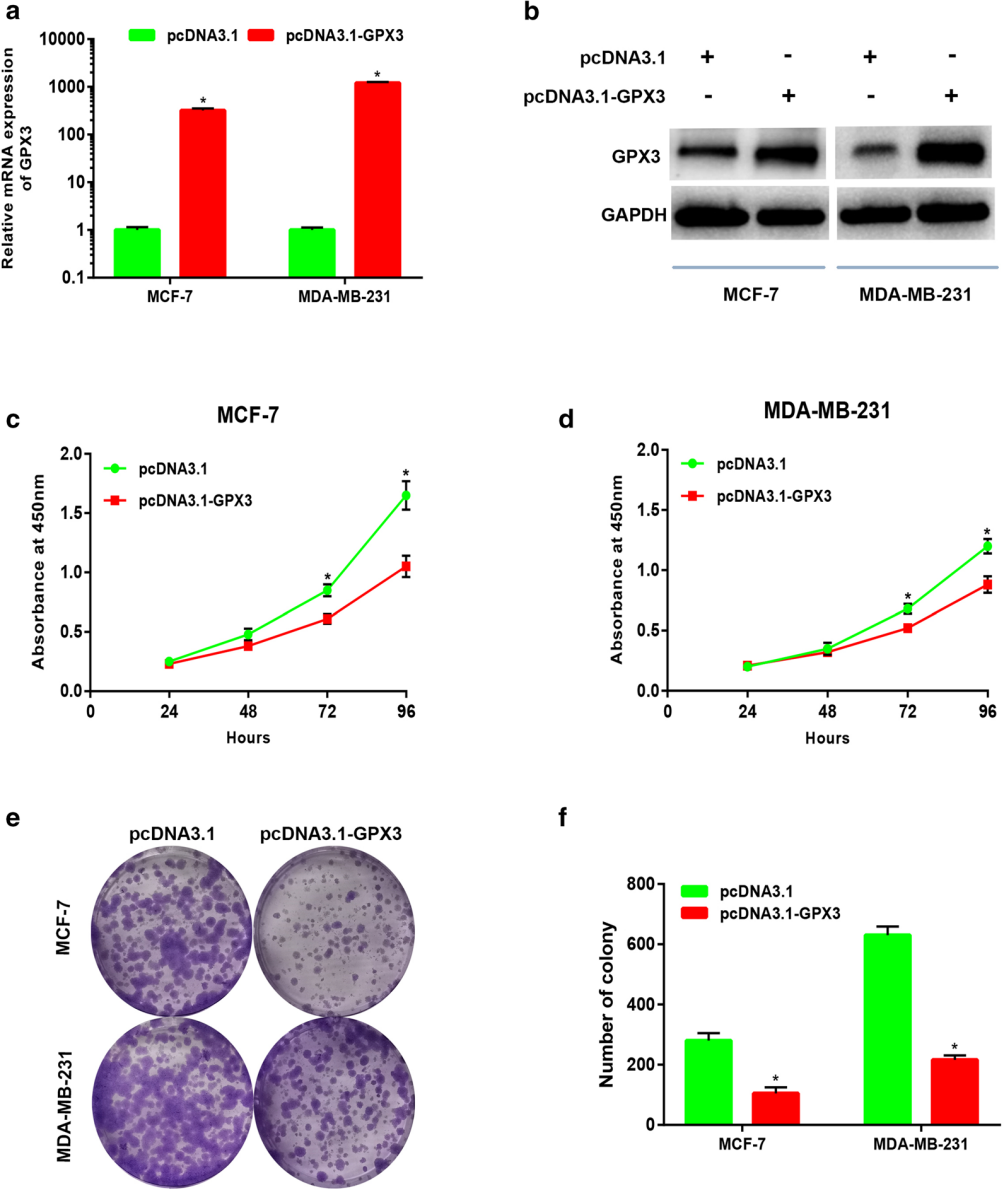
Overexpression of GPX3 inhibited proliferation and colony formation of breast cancer cells in vitro. **a**–**b** The overexpression effect of GPX3-overexpressed plasmid in breast cancer cells. **c**–**d** Overexpression of GPX3 inhibited proliferation of MCF-7 and MDA-MB-231 cells. **e**–**f** Overexpression of GPX3 inhibited colony formation of MCF-7 and MDA-MB-231 cells. *P < 0.05

Given the low expression of GPX3 in breast cancer, overexpression technology was used to study GPX3′s functions. We then constructed the overexpressed plasmid of GPX3. After transfection of GPX3-overexpressed plasmid, GPX3 mRNA and protein expression levels were significantly upregulated in breast cancer cells (Fig. 8a, b). Firstly, we explored the effect of GPX3 on growth of breast cancer cells. CCK-8 assay demonstrated that overexpression of GPX3 markedly suppressed in vitro proliferation of breast cancer cells, MCF-7 and MDA-MB-231 (Fig. 8c, d). Furthermore, colony formation assay also revealed that GPX3 upregulation led to the inhibition of clonogenic capacity of breast cancer cells (Fig. 8e, f). These findings indicated that GPX3 overexpression significantly suppressed in vitro proliferation and colony formation of breast cancer cells.

### GPX3 overexpression inhibited migration and invasion of breast cancer cells

**Fig. 9:**
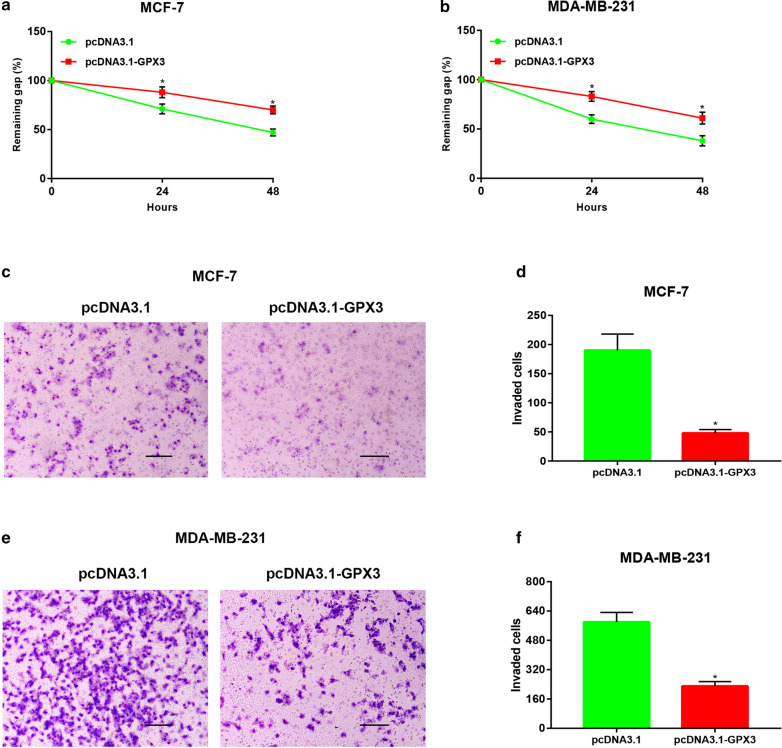
Overexpression of GPX3 suppressed migration and invasion of breast cancer cells in vitro. **a**, **b** Increased expression of GPX3 attenuated migration of MCF-7 and MDA-MB-231 cells. **c**, **d** Increased expression of GPX3 attenuated invasion of MCF-7 cell. **e**, **f** Increased expression of GPX3 attenuated invasion of MDA-MB-231 cell. Bar scale: 150 um; *P < 0.05

Metastasis is another hallmark of malignant tumors, including breast cancer. We intended to ascertain if GPX3 affects metastasis of breast cancer. Wound healing assay was first employed to investigate GPX3′s function in controlling migration of breast cancer cells, and the result demonstrated that overexpression of GPX3 obviously attenuated the migrated ability of breast cancer cells (Fig. 9a, b). Moreover, increased expression of GPX3 could also suppressed invasion of breast cancer cells, which was detected by transwell invasion assay (Fig. 9c–f). Taken together, overexpression of GPX3 suppressed in vitro migration and invasion of breast cancer cells.

### The potential mechanisms responsible for GPX3 downregulation in breast cancer

**Fig. 10:**
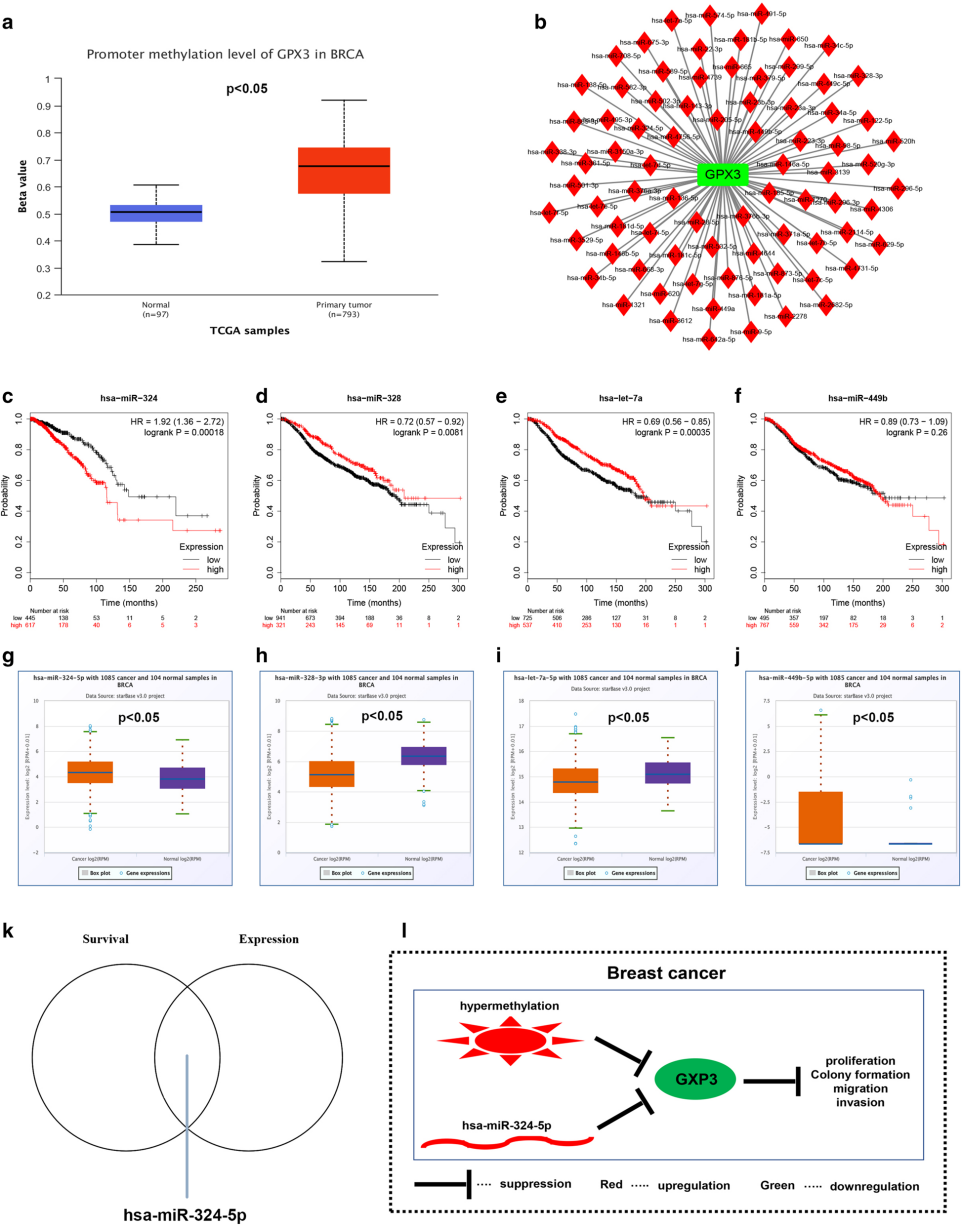
The potential mechanisms responsible for GPX3 downregulation in breast cancer. **a** The promoter methylation level of GPX3 was increased in breast cancer compared with normal controls. **b** The miRNA-GPX3 network. **c**–**f** The prognostic values of four miRNAs in breast cancer. **g**–**j** The expression levels of four miRNAs in breast cancer. **k** The intersection analysis of survival analysis and expression analysis. **l** The model of GPX3′s function and dysregulated mechanism in breast cancer. *P *< 0.05 was considered as statistically significant

Finally, we preliminarily probed the possible molecular mechanisms that accounted for GPX3 downregulation in breast cancer. Promoter hypermethylation may be responsible for expression suppression of tumor suppressors. Intriguingly, we found that the promoter methylation level of GPX3 was significantly upregulated in breast cancer tissues compared with normal controls (Fig. 10a). Gene expression was also frequently negatively regulated by miRNAs at post-transcriptional level. The miRNAs that potentially bind to GPX3 were predicted by starBase database, and 79 miRNAs were finally found. For better visualization, miRNA-GPX3 network was established (Fig. 10b). Based on the action mechanism of miRNA, there should be negative correlation between miRNA and target gene. We performed expression correlation analysis for miRNA-GPX3 pairs. As listed in Table 2, four potential miRNAs (hsa-miR-324-5p, hsa-miR-328-3p, hsa-let-7a-5p and hsa-miR-449b-5p), which were inversely associated with GPX3 expression in breast cancer, were identified. The prognostic values of the four miRNAs in breast cancer were also evaluated by Kaplan–Meier-plotter database (Fig. 10c, d). Survival analysis revealed that, among the four miRNAs, only high expression of hsa-miR-324-5p indicated poor prognosis for patients with breast cancer (Fig. 10c). The expression levels of four miRNAs in breast cancer was subsequently determined by starBase (Fig. 10g–j), and showed that miR-324-5p and hsa-miR-449b-5p were significantly upregulated whereas hsa-miR-328-3p and hsa-let-7a-5p were markedly downregulated in breast cancer compared with normal controls. By combination of survival and expression analysis, miR-324-5p was considered as the most potential upstream miRNA of GPX3 in breast cancer. The above results implied that promoter hypermethylation and miR-324-5p-mediated suppression were two potential mechanisms that may be responsible for GPX3 downregulation in breast cancer (Fig. 10l).

**Table 2:** The expression correlation of GPX3 with predicted miRNAs using TCGA breast cancer data

miRNA	R	P-value
*hsa-miR-324-5p*	*− 0.1790*	*0.0000*
*hsa-miR-328-3p*	*− 0.1570*	*0.0000*
*hsa-let-7a-5p*	*− 0.1480*	*0.0000*
*hsa-miR-449b-5p*	*− 0.1040*	*0.0006*
hsa-miR-629-5p	− 0.0910	0.0026
hsa-miR-4756-5p	− 0.0740	0.0143
hsa-miR-642a-5p	− 0.0680	0.0247
hsa-let-7d-5p	− 0.0670	0.0272
hsa-miR-449a	− 0.0670	0.0275
hsa-miR-589-5p	− 0.0630	0.0368
hsa-miR-181d-5p	− 0.0460	0.1330
hsa-miR-2114-5p	− 0.0460	0.1320
hsa-miR-34a-5p	− 0.0440	0.1490
hsa-miR-449c-5p	− 0.0430	0.1560
hsa-miR-23a-3p	− 0.0410	0.1770
hsa-miR-3150a-3p	− 0.0410	0.1760
hsa-miR-4731-5p	− 0.0410	0.1720
hsa-miR-23b-3p	− 0.0380	0.2070
hsa-miR-491-5p	− 0.0360	0.2310
hsa-miR-4739	− 0.0320	0.2980
hsa-miR-181c-5p	− 0.0290	0.3330
hsa-miR-3612	− 0.0230	0.4560
hsa-miR-582-3p	− 0.0190	0.5300
hsa-miR-650	− 0.0160	0.5940
hsa-let-7b-5p	− 0.0120	0.6960
hsa-miR-338-3p	− 0.0090	0.7600
hsa-miR-2278	− 0.0080	0.7840
hsa-miR-122-5p	− 0.0060	0.8390
hsa-miR-181a-5p	− 0.0040	0.9080
hsa-miR-181b-5p	− 0.0030	0.9180
hsa-miR-3139	− 0.0030	0.9120
hsa-miR-4644	− 0.0030	0.9290
hsa-miR-501-3p	− 0.0020	0.9540
hsa-miR-2682-5p	− 0.0020	0.9590
hsa-miR-4306	− 0.0010	0.9770
hsa-miR-9-5p	0.0000	0.9870
hsa-miR-620	0.0000	1.0000
hsa-miR-1321	0.0000	1.0000
hsa-miR-98-5p	0.0050	0.8600
hsa-let-7e-5p	0.0080	0.7830
hsa-miR-185-5p	0.0100	0.7330
hsa-miR-1270	0.0140	0.6380
hsa-let-7 g-5p	0.0160	0.5880
hsa-miR-205-5p	0.0170	0.5790
hsa-miR-371a-5p	0.0210	0.4840
hsa-miR-873-5p	0.0230	0.4530
hsa-miR-34b-5p	0.0310	0.3040
hsa-miR-532-5p	0.0410	0.1780
hsa-miR-296-3p	0.0410	0.1810
hsa-miR-708-5p	0.0430	0.1570
hsa-miR-3529-5p	0.0510	0.0901
hsa-miR-574-5p	0.0570	0.0612
hsa-miR-876-5p	0.0610	0.0461
hsa-miR-296-5p	0.0680	0.0260
hsa-miR-502-3p	0.0750	0.0139
hsa-miR-136-5p	0.0820	0.0067
hsa-miR-28-5p	0.0870	0.0040
hsa-miR-361-5p	0.0870	0.0043
hsa-miR-520 h	0.0940	0.0020
hsa-miR-668-3p	0.0980	0.0012
hsa-miR-520 g-3p	0.1110	0.0002
hsa-miR-376b-3p	0.1190	0.0001
hsa-miR-675-3p	0.1460	0.0000
hsa-let-7f-5p	0.1500	0.0000
hsa-miR-34c-5p	0.1500	0.0000
hsa-miR-665	0.1530	0.0000
hsa-miR-138-5p	0.1550	0.0000
hsa-miR-146a-5p	0.1610	0.0000
hsa-miR-376a-3p	0.1680	0.0000
hsa-miR-22-3p	0.1990	0.0000
hsa-miR-299-5p	0.2110	0.0000
hsa-let-7i-5p	0.2500	0.0000
hsa-miR-495-3p	0.2600	0.0000
hsa-miR-143-3p	0.2670	0.0000
hsa-miR-889-3p	0.2670	0.0000
hsa-miR-146b-5p	0.2830	0.0000
hsa-miR-379-5p	0.3090	0.0000
hsa-miR-223-3p	0.3690	0.0000
hsa-let-7c-5p	0.3720	0.0000

## Discussion

Breast cancer is the most common cancer type in women. The molecular mechanism of carcinogenesis of breast cancer is still unclear and need to be further investigated. Increasing findings have showed that GPXs are critical regulators in onset and progression of human cancer. However, the knowledge of GPXs in breast cancer is still limited.

ROC curve and survival analysis for GPXs family revealed that some of them might serve as promising diagnostic and prognostic biomarkers for breast cancer, especially GPX2 and GPX3. Expression analysis demonstrated the significant low expression of GPX3 in breast cancer. GPX3 was reported to act as a tumor suppressor in human cancer. For example, Cai et al. indicated that GPX3 prevented migration and invasion of gastric cancer by targeting NF-kB/Wnt5a/JNK signaling [20]; Lee et al. suggested that GPX3 arrested cell cycle and functioned as a tumor suppressor in lung cancer [21]; Hua et al. showed that silencing GPX3 expression promoted tumor metastasis in human thyroid cancer [22]; Caitlyn et al. revealed that plasma GPX3 limited the development of colitis -associated carcinoma [23]. However, the function and mechanism of GPX3 in breast cancer have not been reported and need to be further elucidated.

Next, we confirmed the low expression of GPX3 in breast cancer cells and tissues using qRT-PCR, western blot and IHC, which supported the results of bioinformatic analysis. Functional experiments revealed that overexpression of GPX3 significantly inhibited in vitro proliferation, colony formation, migration and invasion of breast cancer cells.

Previous studies have showed the effect of promoter methylation level in regulating gene expression [24]. Thus, we preliminarily evaluated the promoter methylation level of GPX3 in breast cancer, and found that it was significantly upregulated in breast cancer compared with normal breast tissues. Moreover, Mohamed et al. also demonstrated the link between promoter hypermethylation of GPX3 and inflammatory breast carcinogenesis [25]. The report together with our finding revealed that hypermethylation of GPX3 promoter might be a potential mechanism responsible for GPX3 downregulation in breast cancer.

miRNAs are involved in multiple biological processes by suppressing gene expression [2, 26–28]. We also explored the upstream regulatory miRNAs of GPX3. By combination of correlation analysis, survival analysis and expression analysis for these miRNAs, miR-324-5p was regarded as the most potential miRNA, which was overexpressed, negatively correlated with GPX3 expression, and possessed poor prognosis in breast cancer. Numerous studies have demonstrated that miR-324-5p served as an oncogenic miRNA in human cancer. For example, miR-324-5p promoted progression of papillary thyroid carcinoma via microenvironment alteration [29]; miR-324-5p facilitated progression of colon cancer by activating Wnt/beta-catenin pathway [30]. Moreover, the relationship between GPX3 and miR-324-5p has already been reported in lung cancer [31]. Thus, overexpressed miR-324-4p might be another mechanism that accounted for GPX3 downregulation in breast cancer. In the future, the oncogenic roles of miR-324-5p need to be further investigated by in vitro and in vivo assays.

## Conclusions

In summary, our current findings indicate that GPX3 is markedly downregulated in breast cancer, promotes in vitro growth and metastasis of breast cancer cells, and servers as a promising diagnostic or prognostic biomarker for patients with breast cancer. Moreover, we also elucidate that promoter hypermethylation and miR-324-5p-mediated suppression may be two potential mechanisms responsible for GPX3 downregulation in breast cancer. These results provide key clues for developing effective therapeutic targets and biomarkers for breast cancer.

## Data Availability

The data in this work are available from the corresponding author on reasonable request.
